# Granulicatella adiacens Endocarditis of a Bioprosthetic Aortic Valve

**DOI:** 10.7759/cureus.40720

**Published:** 2023-06-21

**Authors:** Amanda Warren, Adina Amin, Jude Elsaygh, Kevin Pink

**Affiliations:** 1 Internal Medicine, NewYork-Presbyterian Brooklyn Methodist Hospital, Brooklyn, USA

**Keywords:** infective endocarditis, culture-negative infective endocarditis, fastidious bacteria, infectious disease medicine, infectious disease, adult cardiology, bioprosthetic aortic valve, granulicatella adiacens

## Abstract

Infective endocarditis (IE) is relatively uncommon; however, when it is diagnosed, it is usually among those with known cardiac valvular abnormalities. The most common pathogens that cause endocarditis are streptococci (mainly viridans), enterococci, and other streptococci species. An extremely rare pathogen that could cause IE is *Granulicatella*. This gram-positive coccus classically inhabits human mucosal surfaces and only rarely causes disease. We present an incredibly rare case of a 74-year-old female with a bioprosthetic aortic valve replacement, who presented with headache and weakness and was subsequently found to have recurrent *Granulicatella adiacen*s infective endocarditis.

## Introduction

Infective endocarditis (IE) is an infection of the endocardial surfaces of the heart and typically involves the colonization of the cardiac valves with virulent microorganisms. Some of the most common pathogens that cause endocarditis are streptococci (mainly viridans), enterococci, and other streptococci species. Some less common variants that still remain well known to clinicians are the following organisms: *Haemophilus* species, *Aggregatibacter* species, *Cardiobacterium* species, *Eikenella corrodens*, and *Kingella* species (HACEK). An extremely rare pathogen that has the potential to cause IE is *Granulicatella adiacens. *This gram-positive coccus is a nutritionally variant streptococcus that classically inhabits human mucosal surfaces and only rarely causes disease [1]. *Granulicatella adiacens* is estimated to account for 5%-6% of the cases of infective endocarditis worldwide [2].

## Case presentation

A 74-year-old female with a past medical history of nonobstructive coronary artery disease, hypertension, hyperlipidemia, osteoarthritis, and severe aortic stenosis with remote bioprosthetic aortic valve replacement presented to the emergency department with headache and transient right-sided weakness. A stroke code was initiated on arrival at the hospital, and the patient was found to have a small left frontoparietal convexal subarachnoid hemorrhage. A cerebral angiogram was performed, which was unremarkable for an underlying aneurysm or other vascular lesions that would explain the patient’s subarachnoid hemorrhage. Echocardiography was performed, which showed an ejection fraction of 65% with a mildly dilated atrium and elevated transvalvular gradient across the prosthetic aortic valve with an acceleration time of 130 ms. The patient’s blood cultures collected on admission began growing gram-positive bacteria, and she was started on vancomycin and cefepime. Six days after cultures were collected, they speciated, showing *Granulicatella adiacens* bacteremia. The patient was advised to start gentamicin therapy but refused due to concern for renal injury and was subsequently treated with intravenous ceftriaxone. A transesophageal echocardiogram was obtained, which indicated an ejection fraction of 60%-65% with the thickening of the prosthetic aortic valve resulting in severe aortic stenosis. The aortic valve area by planimetry average area was 1.0 cm^2^ with the velocity and mean gradient both markedly elevated. The elevated gradient was possibly due to the high flow state in the setting of the present infection. The patient also was noted to have a suspected aortic root abscess present.

**Figure 1:**
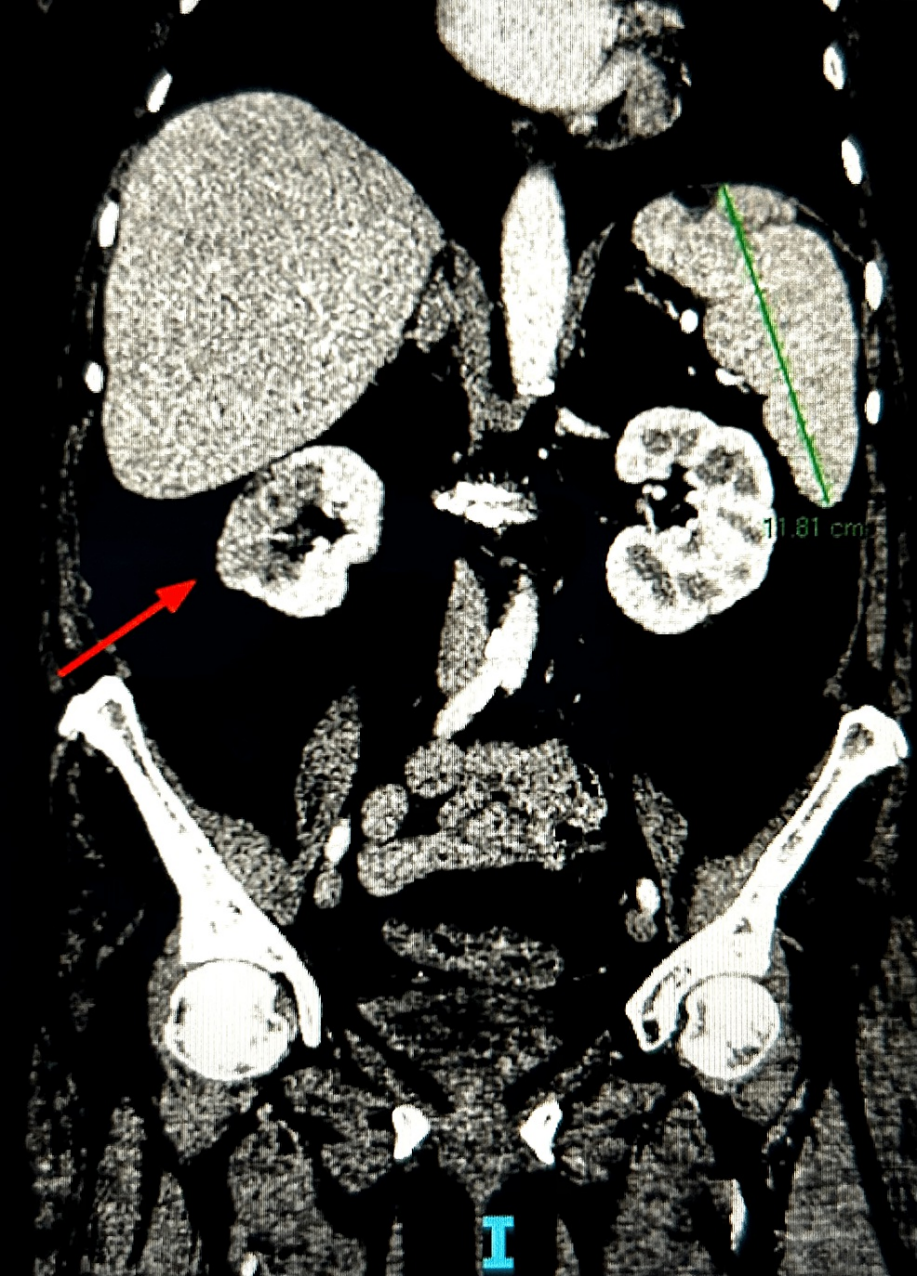
Contrast-enhanced computed tomography of the abdomen and pelvis with the red arrow indicating right wedge-shaped renal infarction.

To further evaluate the abscess, a gallium scan was obtained, which did not show cardiac uptake, so cardiothoracic surgery decided to continue with conservative management at that time. The patient was treated with intravenous ceftriaxone for a total of six weeks. After the completion of the antibiotic course, the patient later returned to the emergency department with complaints of left-sided flank pain. The computed tomography imaging of the abdomen and pelvis with intravenous contrast showed a wedge-shaped infarct in the right kidney, which was concerning for ischemia or an embolic phenomenon (see Figure 1).

On subsequent readmission to the hospital, the patient’s blood cultures were once again positive for *Granulicatella adiacens*, which was concerning for recurrent endocarditis. The patient underwent re-operative aortic valve repair with modified Nicks root enlargement. Valvular culture sent after the procedure was also positive for *Granulicatella adiacens*. The patient was then treated with intravenous gentamicin and vancomycin for an additional six weeks with proper clearance of the bacteria.

## Discussion

*Granulicatella adiacens* is a gram-positive, anaerobic bacterium that is a rare cause of endocarditis. It is part of the normal flora of the oral cavity and upper respiratory tract but can cause infections in immunocompromised individuals, especially those with underlying cardiac disease. Although endocarditis caused by *G. adiacens* is uncommon, it is important to recognize and treat it promptly due to the risk of serious complications. If not managed properly, IE due to *G. adiacens* can result in valvular damage, heart failure, embolization, cerebral mycotic aneurysm, and the extension of vegetations into the wall of the atria [3].

Diagnosing *Granulicatella adiacens* infection can be challenging, as the organism is a slow-growing, fastidious bacterium that is difficult to culture. Blood cultures remain the gold standard for diagnosis, and multiple sets of blood cultures should be obtained to increase sensitivity. In addition, serological tests such as enzyme-linked immunosorbent assay (ELISA) and polymerase chain reaction (PCR) can aid in diagnosis. Molecular techniques such as 16S rRNA gene sequencing and matrix-assisted laser desorption/ionization-time of flight mass spectrometry (MALDI-TOF MS) can also be used for identification [3,4]. Echocardiogram also plays a critical role in the evaluation and management of infective endocarditis. Echocardiograms are utilized to detect valvular vegetations and assess the severity of valvular dysfunction and are useful in the assessment of abscesses. The patients with mechanical valves and *G. adiacens* endocarditis are at an increased risk for paravalvular leaks and dehiscence [5]. Blood cultures, PCR, and echocardiograms are all diagnostic modalities that can aid in the prompt and accurate diagnosis of *G. adiacens* infection. Early diagnosis allows for the acute initiation of appropriate antimicrobial therapy and improves patient outcomes.

The choice of antimicrobial therapy for *G. adiacens* endocarditis is based on the antimicrobial susceptibility of the organism and the severity of the infection. Given the rarity of this infection, there are no specific guidelines for the treatment of *G. adiacens* endocarditis. However, a combination of penicillin or ceftriaxone with an aminoglycoside is often used as initial therapy. In patients with a penicillin allergy, vancomycin or clindamycin can be used as an alternative. The duration of treatment is typically 4-6 weeks but may be extended in patients with complications such as embolic events or persistent bacteremia. Additional studies show a similar duration of success as seen in a study performed by Murdoch et al. (2009), in which 18 patients with *G. adiacens* endocarditis were successfully treated with a combination of penicillin and gentamicin for a median of 42 days [6]. The close monitoring of patients with *G. adiacens* endocarditis is essential to ensure adequate response to therapy and to detect any potential complications that may require intervention.

## Conclusions

Although a rare cause of seronegative infective endocarditis, *Granulicatella adiacens* should be considered in patients with clinical or echocardiographic evidence of infective endocarditis. Due to the fastidious nature of *G. adiacens*, it can lead to delayed diagnosis and often result in delayed treatment initiation. Current treatment recommendations for *G. adiacens* infective endocarditis include the intravenous administration of ceftriaxone, penicillin, or vancomycin most commonly in combination with an aminoglycoside. Not all isolates of *G. adiacens* are susceptible to ceftriaxone, and the utilization of a concomitant aminoglycoside may be essential for proper treatment. Our patient’s failed treatment with intravenous ceftriaxone highlights the importance of collecting sample speciation, the use of serological testing, and molecular techniques in treating rare causes of infective endocarditis to ensure the proper eradication of the causative pathogen.
